# Organising for Political Advocacy—the Case of the *Netzwerk Solidarische Landwirtschaft*

**DOI:** 10.1007/s10767-025-09537-1

**Published:** 2025-07-28

**Authors:** Leonie Guerrero Lara, Baldur Kapusta, Jessica Duncan, Giuseppe Feola

**Affiliations:** 1 Copernicus Institute of Sustainable Development, Utrecht University, Princetonlaan 8, 3584CB Utrecht, the Netherlands; 2 Netzwerk Solidarische Landwirtschaft, Mittelstr. 1, 51149 Köln, Germany; 3 Rural Sociology Group, Wageningen University & Research, Droevendaalsesteeg 4, 6708 PB Wageningen, Netherlands

**Keywords:** Social movements, Agri-food movements, Interest groups, Strategies, Sustainable agriculture, Community-supported agriculture, Germany

## Abstract

This article analyses the efforts by the German Community-Supported Agriculture (CSA) network to carry out political advocacy work as a strategy to strengthen the movement and induce change in the industrial agri-food system—a strategy which thus far has been omitted by studies on CSA. To address this gap, we build on the literature on social movements and advocacy groups. Drawing on experiences with political advocacy between 2018 and 2022, this article maps enabling factors and barriers for advocacy as well as the related internal debates and contestations. We find that the organisational structure of the network (or in some instances, the lack thereof) influences strategies and tactics, resources, emotions and group dynamics, and advocacy spaces. The study concludes that advocates should pay due attention to their movement’s organisational structure in relation to advocacy, including potential tensions around member participation in decision-making, responsibilities, and legitimacy of advocates. Attention should also be paid to the visibility and valorisation of advocacy work, and (mis-)matches between the internal structure and advocacy spaces.

## Introduction

That we were mentioned in the government agreement of the grand coalition, that was an incredible success! [The network] was the only agricultural association that was [explicitly] mentioned in the government agreement. Just imagine! And I would say that many people [within the network] didn’t get that. And what follows from this, namely, that we could have built on this. (Interview #1)

In 2018, the *Netzwerk Solidarische Landwirtschaft*,^1^ the national German network of Community-Supported Agriculture (CSA), a growing, but still considerably small and niche agricultural grassroots movement, was mentioned in the national-level government agreement: *‘*Within the scope of lighthouse projects, we want to promote and support best practice examples of regional value chains and marketing, e.g. the Netzwerk Solidarische Landwirtschaft’ (CDU/CSU & SPD, 2018).

The promise of institutional support for the German CSA network (hereafter: SOLAWI) appeared remarkable since the Ministry for Food and Agriculture was led by the Christian Democratic Union (CDU), a conservative party whose agricultural policy caters largely to the interests of the German Farmers’ Association (Deutscher Bauernverband e. V.—DBV) (Feindt, 2009). The DBV, representing 90% of all farmers in Germany, advocates mainly for productivist measures that benefit large and medium-sized farms (Feindt, 2009). In contrast, SOLAWI was born as a bottom-up response to the industrial, globalised agri-food system, which forces smallholders to grow and industrialise, as otherwise, they are squeezed out of the market (Blättel-Mink et al., 2017). Seeking to bring about a paradigm change towards a regional, ecologically sound, and socially responsible agriculture, SOLAWI promotes the spread of a CSA model, which isolates small-scale producers from market pressures: establishing a long-term producer–consumer partnership in which consumers collectively share the risks and costs of farming in return for a harvest share (Rommel et al., 2022).

The explicit naming of SOLAWI in the government agreement was the result of the persistent and diligent political advocacy work of a couple of activists who used their personal contacts with Members of Parliament (MPs) across different parties to make a case for the CSA network (I-2).^2^ However, what first appeared to be an opportunity led to internal contestations and ultimately contributed to the disengagement of several members who were actively pushing for political advocacy as a key strategy of the network.

Political advocacy refers to the ‘efforts to push public policy in a specific direction on the behalf of constituencies or a general political idea’ (Beyers et al., 2008, 1106). It is pursued via influencing policy making, legislation, political parties, and state bureaucracies (Amenta et al., 2010). As such, it represents a strategy for social movements to bring about societal change by trying to work from ‘within’ a system (Beyers et al., 2008). The role of political advocacy as a strategy for SOLAWI has been repeatedly debated internally: (How much) does the network want to prioritise political advocacy? What does this mean for their resource allocation? Who is a legitimate advocate? Is political advocacy a viable strategy for stabilising the movement and contributing to agri-food system transformations, or will the institutional support backfire and make SOLAWI become dependent on politicians and lose its core values and vision?

Scholarship on CSA, including in the German context, has paid little attention to this topic. Among the few publications that have addressed institutional upscaling in the context of CSA networks, the most comprehensive study was carried out by Bonfert (2022). Drawing on the experience of the UK CSA network,^3^ he describes a strategic shift from covering advocacy work via alliance building towards pro-active advocacy work on their own behalf. According to Bonfert, mobilising resources and appointing a policy coordinator for the network enabled the UK CSA network to issue policy briefings, raise awareness among politicians about the potential of CSAs, and articulate national policy demands (ibid.).

(Scholar-)activist contributions have further cursorily engaged with transnational advocacy work for decentralised food chains (Hitchman, 2014; Stapleton, 2019). These contributions have highlighted tactics such as capacity-building workshops, international exchange on advocacy work, building alliances with other international organisations, and engaging in policy spaces that are organised around the participation of different stakeholders, including civil society and grassroots movements (Hitchman, 2014; Stapleton, 2019). In sum, whilst some studies have acknowledged attempts of CSA networks to influence policy making, a systematic analysis of political advocacy in such networks, including its enabling and inhibiting factors, is still lacking.

In this paper, we address this gap by taking stock of and reflecting on past attempts to advocate for CSA and the resulting internal debates and contestations within SOLAWI. Specifically, we analysed what enabled or hindered political advocacy work between 2018 and 2022 in order to examine political advocacy as a strategy for SOLAWI. For this purpose, we draw on the literature relating to social movements and advocacy groups.

This paper primarily makes an empirical contribution that aims to inform advocacy work, while also offering scholars insights into poorly understood organisational dynamics and a movement that has been overlooked in the political advocacy literature. In doing so, this paper also brings the role of emotions into analyses of political advocacy, which have been dominated by structural theories, thus offering an enriched perspective—crystallised into a framework—which promises to have wider applicability in political advocacy research.

## Political Advocacy Within SOLAWI

SOLAWI was founded in 2011 by a number of CSA farmers as well as by activists with backgrounds in the anti-globalisation, solidarity economy, and right-to-food movements. Since its foundation, the movement has grown considerably, from 12 initial members to over 431^4^ individual CSA initiatives (with an additional 99 initiatives in foundation). The network provides a space for mutual exchange and learning for CSA initiatives and reconciles positions among its diverse members. Indeed, it brings together an array of various types of CSA initiatives—including producer-led initiatives, consumer-led initiatives, gardening collectives, peasant family farms, anti-capitalist-emancipatory efforts, and anthroposophical initiatives.

**Fig. 1:**
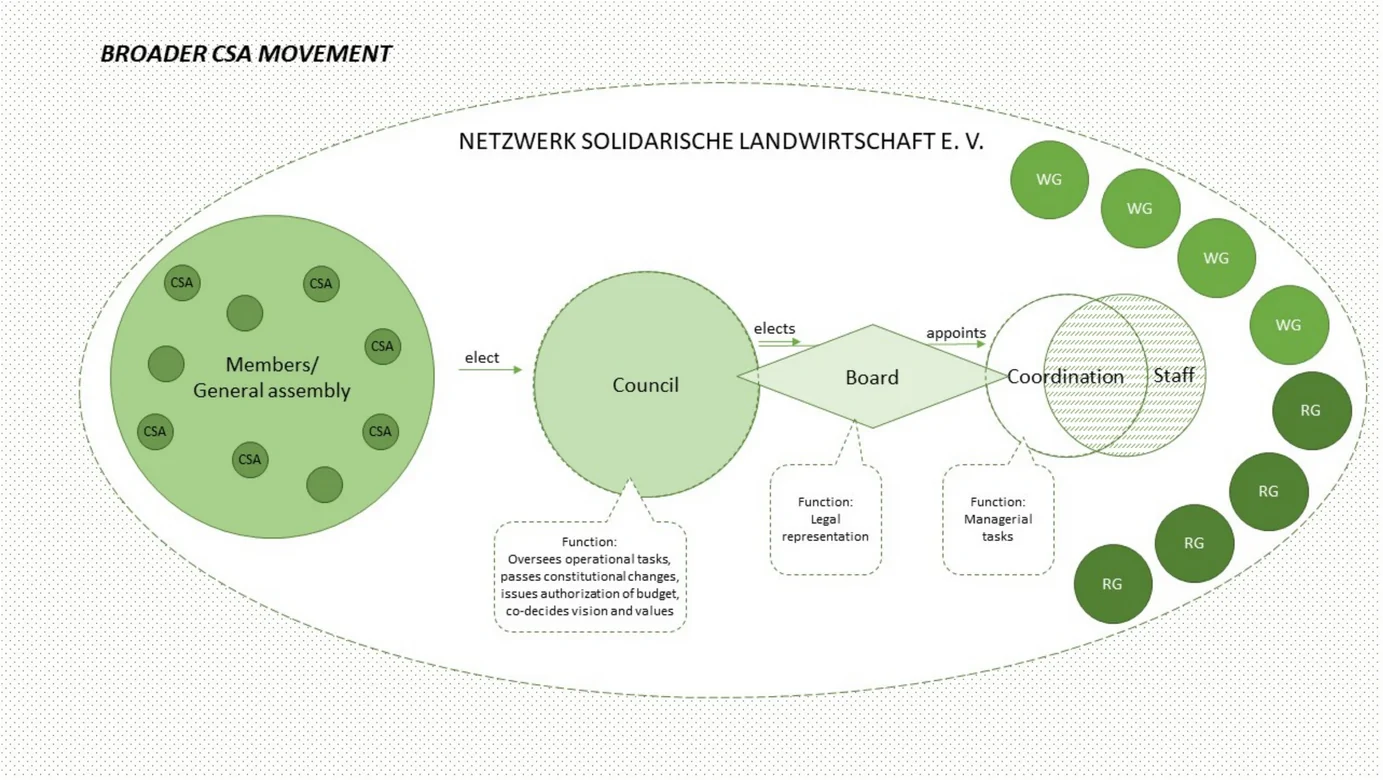
Organisational diagram of the Netzwerk Solidarische Landwirtschaft e. V. Adapted from: https://www.solidarische-landwirtschaft.org/das-netzwerk/ueber-uns/struktur. Abbreviations: WG = working group; RG = regional group

Formally organised as an association, the network is composed of four different organs: the general assembly, the council, the coordination, and the board (Fig. 1). On a bi-yearly basis, members elect the council, which represents the interests of all members and is involved in the development of the network’s vision, goals, and values (NWSL, 2023). Moreover, the network has several decentralised groups, including regional groups for promoting exchange between nearby CSA initiatives and working groups which span numerous topics, from societal transformations to self-organised vocational training. In 2022, approximately 50 people were actively engaged in different spaces of the network. As a ‘grassroots democracy’, the network aspires to make decisions within the different organs and working groups in line with sociocratic principles based on either consent or consensus.

The proclaimed goal of the network consists of safeguarding and promoting a sustainable peasant agriculture based on a close consumer–producer partnership that views agriculture as a joint responsibility. This goal implies fundamentally transforming the industrial agri-food system (NWSL n.d.). The network pursues this goal via a variety of activities, including political advocacy work.

**Fig. 2:**
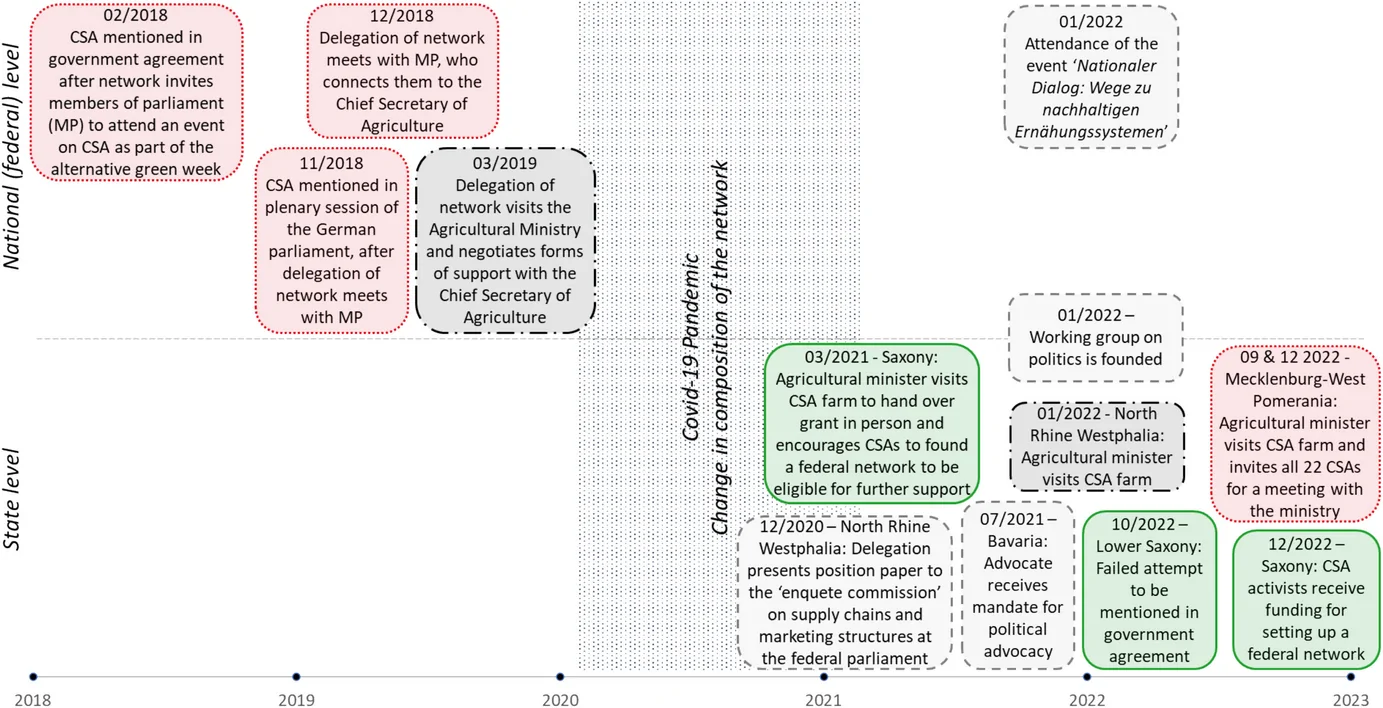
Own creation. The timeline outlines different political advocacy activities across the national (federal) and state levels. The colour and line type of the shape symbolise the actor involved; red dots: Social Democrats; black, long dashes and dots: Christian Democrats; green solid line: Green Party; grey dashes: non-party related activities

In the past, SOLAWI has carried out political advocacy in various ways and by various activists, on both the national (federal) and state levels (see Fig. 2). A major achievement of these advocacy efforts was the aforementioned reference to CSA in the government agreement in 2018.

The outbreak of the COVID-19 pandemic at the beginning of 2020 brought advocacy activities by and large to a halt. Additionally, at the beginning of 2021, several key advocates decided to disengage from SOLAWI after not being re-elected as council and board members. As a result, the network was left without active members with expertise in conducting political advocacy, and the network’s social ties to politicians were significantly weakened. The subsequent rebuilding of political advocacy was largely driven by CSA initiatives which, in collaboration with SOLAWI and the newly found working group on politics, invited agricultural ministers from their respective federal states to their CSA farm, introducing them to the CSA model and its potential to reinvent agriculture.

## A Framework for Understanding Political Advocacy

The framework presented in this section was built by way of an iterative process that led from exploratory fieldwork and data collection, engagement with literature on political advocacy, and data analysis to the refining of final concepts and dimensions of political advocacy relevant and useful for the scope of this study.

After finalising the data collection, we inductively coded the interviews and data sources (see ‘Research Design’). Building on this first round of data analysis, we conducted a selective literature review to identify key concepts from the literature on political advocacy and assessed whether they matched the empirical case at hand. While conducting the literature review, we noticed a stark fragmentation of the field, which spans scholarly traditions ranging from social movement studies to sociology to economics to political science (Andrews & Edwards, 2004; Beyers et al., 2008). This fragmentation is reflected by the various labels for groups pursuing questions of advocacy, such as interest or pressure groups, advocacy organisations, non-profit organisations, non-governmental organisations (NGOs), social movement organisations, and civil society organisations, to name a few (Andrews & Edwards, 2004; Beyers et al., 2008; Císař, 2013). To date, exchange between different disciplines and strands of literature have remained cursory, rendering it challenging to study advocacy (Beyers et al., 2008). Therefore, we used our insights from the first round of data analysis and experiences of working with different agri-food movements to select those strands of advocacy literature that were most appropriate to apply to the empirical case at hand, including considering some additional aspects not covered directly by these perspectives (see ‘Emotions and group dynamics’). Because of the limited scope of this paper, we focus primarily on the internal dynamics within SOLAWI and do not systematically assess the role of broader political opportunities (for a treatment of political opportunities see MacIndoe & Beaton, 2019 more generally; and Shawki, 2010 in the context of food movements).

Our framework is constituted by five dimensions pertaining to the analysis of political advocacy: strategic orientation, organisational structure, resources, places and spaces of advocacy, and emotions and group dynamics that can enable (or hinder) political advocacy. As shown in Table 1, these dimensions allow us to explore the feasibility and desirability of political advocacy from various angles. We now briefly outline the relevance of each of these five dimensions.

**Table 1:** Framework: dimensions of political advocacy

Dimension	Guiding questions
Strategies and tactics	What long-term strategy does the network have? What advocacy tactics are prioritised: outsider or insider tactics? What position does the network have towards insider tactics in general?
	Almog-Bar and Schmid (2014); Beyers et al. (2008); Giugni and Grasso (2018); Chubin (2020); Doherty and Hayes (2019)
Organisational structures	How professionalised and formalised is the network? Is the degree of professionalisation conducive for political advocacy? How is decision-making organised? What influence do the network’s members have on advocacy?
	Andrews and Edwards (2004); Giugni and Grasso (2018); Almog-Bar and Schmid (2014); Chewinski and Corrigall–Brown (2020); Foley and Edwards (2002); Flavell (2023)
Resources	What material (i.e. financial and physical), human, moral, and socio-organisational resources has the network mobilised for political advocacy? On human resources specifically, do advocates have the necessary skills, abilities, and professional experience for advocating?
	Andrews and Edwards (2004); Edwards and McCarthy (2004); Chewinski and Corrigall–Brown (2020); Almog-Bar and Schmid (2014); Mosley, (2010; 2011)
Emotions and group dynamics	What positive (or negative) affective emotions have hindered or enabled advocacy within the network?
	Flam et al. (2005); Jasper (2011); Goodwin & Jasper (2006); Scoping fieldwork
Advocacy spaces	In what spaces (supranational, national (federal), state, or communal) was advocacy work undertaken? (How) does this match the structure of the political system?
	Giugni &and Grasso (2018); Armingeon (2002)

## Strategies and Tactics

Movements pursue different strategies and tactics to realise their goals. The term ‘strategies’ refers to the various decisions made in the interest of a social movement or its constituent organisations (Meyer & Staggenborg, 2021; Smithey, 2009). They typically denote long(er)-term thinking, which connects the means and ends of collective action (Doherty & Hayes, 2019). This fundamentally entails choices about ‘tactics,’ which form the ‘essence of collective action’ (Ennis, 1987, 520). Tactics include protests, boycotts, petitions to civil disobedience, commemorations, and political advocacy and are advanced to persuade, encourage negotiation, and invite different responses from a range of actors (Smithey, 2009).

While diverse classification of social movement strategies and tactics co-exist, scholars interested in advocacy work typically distinguish between insider and outsider tactics (Almog-Bar & Schmid, 2014; Beyers et al., 2008; Giugni & Grasso, 2018). The former consist of working ‘inside the system’ via institutional tactics such as networking and advocating with political and administrative elites (Beyers et al., 2008). The latter refer to extra-institutional tactics that intend to induce change by working ‘outside the system’—for instance, via protests, manifestations, direct action, mass media, and campaigns (Giugni & Grasso, 2018) as well as by creating spaces for experimentation and prefiguration (Yates, 2020).

Research has shown that movements and interest groups, including farmer associations, often combine insider tactics, such as direct access to politicians and governmental authorities, with outsider tactics; for example, they may engage in manifestations and work to be ‘vocal in the public sphere’ (Beyers et al., 2008; Binderkrantz, 2005; Nicolosi et al., 2025).

The choice of tactics is typically influenced by the maturity of the movement. Outsider tactics are prevalent in the nascent phase of social movements since they contribute to their organisational maintenance and member recruitment, while older and more stable movements tend to increasingly utilise insider tactics (Beyers et al., 2008). Over their lifetime, movements therefore (re-)negotiate and balance the associated (dis-)advantages of the respective tactics. For instance, insider tactics enable access to decision-makers and thus can contribute to policy change (Almog-Bar & Schmid, 2014). At the same time, movements that utilise insider tactics can become dependent on state resources and thus become more prone to being co-opted.

## Organisational Structure

Various scholars (e.g. Andrews & Edwards, 2004) have highlighted the role of the organisational structure for its capacity or likelihood to engage in advocacy work. This capacity typically relates to the movement’s organisational size, degree of professionalisation and formalisation, and decision-making structure (Almog-Bar & Schmid, 2014; Giugni & Grasso, 2018).

A key metric for assessing the organisational size and the professionalisation of a movement is the number of paid staff members.^5^ Larger organisations tend to have more staff members, which is positively correlated with a movement’s likelihood to engage in political advocacy (Almog-Bar & Schmid, 2014). Staff members play a vital role since they can ‘aggregate privately held resources for collective purposes [such as advocacy work] from socially dispersed individuals’ (Andrews & Edwards, 2004, 489). Furthermore, staff members tend to spur the professionalisation of movements. Research has shown that this process often goes hand-in-hand with insider strategies, including advocacy work. Typically, the professionalisation of a movement is also accompanied by its formalisation, which is manifested by adopting a formal legal status and written constitution (Giugni & Grasso, 2018). At the same time, processes of professionalisation may risk ‘alienating advocates from memberships and constituencies’ (Onyx et al., 2010, 46). In sum, the literature on advocacy argues that small organisations tend to be less institutionalised, formal, and bureaucratic, that they have fewer obligations to governmental agencies, and that they engage less often in advocacy than larger organisations (Almog-Bar & Schmid, 2014).

Decision-making mechanisms are another key dimension of the organisational structure of movements. Members co-decide or at least influence the strategies and tactics of a movement, and their role in the movement’s governance shapes how, to what extent, and on what topics advocacy work is carried out (Foley & Edwards, 2002). However, decision-making can be organised in different ways, ranging from so-called ‘oligarchies’, where members have little to no power over the board, to decentralised grassroots democracies where members jointly decide most aspects of the movement’s life (ibid.).

## Resources

The mobilisation of resources is key for the initiation and continuation of political advocacy work. In this article, we distinguish four types of resources: material, human, social-organisational, and moral resources (adapted from Edwards & McCarthy, 2004). Among material resources, financial resources are vital for advocacy work since they are a prerequisite for hiring additional staff members to carry out advocacy work and ‘may contribute to a general aura of power and prestige around the organization, increasing political clout and making policy makers more responsive to their overtures’ (Mosley, 2011, 443). Additionally, some studies suggest that financially stable movements invest more in political advocacy work than underfunded movements (Almog-Bar & Schmid, 2014).

Building on the above-described trends associated with staffing movements, the literature also shows that human resources are important for political advocacy. These resources include labour provided by paid staff and volunteers as well as the skills, experience, and expertise of movement members (Edwards & McCarthy, 2004). Since political advocacy is highly time-consuming and often extends beyond regular work hours, advocates need to be willing to make sacrifices and invest their own time for the ‘good cause’ (Almog-Bar & Schmid, 2014). While few scholars would contest that advocates require a specific skill set for conducting advocacy work, such as superior communication skills, being politically savvy, and seizing opportunities for advocacy (Mosley, 2011), this domain has only been marginally explored (Almog-Bar & Schmid, 2014). Scholars have furthermore pointed out that not all advocates possess the necessary capabilities, specific skillsets, or expert knowledge to enter political arena. This lack of knowledge and skills can be explained by the fact that advocacy significantly differs from the other day-to-day tasks of movement members or employees (Almog-Bar & Schmid, 2014), which makes attending special trainings necessary (Mosley, 2010).

Social movement scholars have furthermore highlighted the importance of social-organisational resources such as ‘infrastructures, social ties and networks, affinity groups, and coalitions’ (Edwards et al., 2019, 81). Socio-organisational resources are of particular relevance for the study of advocacy work since network relations can help sustain advocacy work by providing access to resources and since coalitions are key for coordinating advocacy with other actors (Andrews & Edwards, 2004).

Finally, outsiders such as researchers, politicians, and celebrities can provide moral resources to movements by enhancing their legitimacy or by openly proclaiming solidarity and sympathetic support (Edwards et al., 2019). Positive media coverage can further legitimise and mobilise support for movements (Pilny et al., 2014). Building on an empirical analysis of several agricultural advocacy groups in Germany, Feindt (2009) and Kleinschmit and Feindt (2004) found that the frequency of media coverage and the image of agricultural advocacy groups in the media (i.e. whether the group is portrayed as part of the problem or part of the solution) are relevant indicators for the advocacy group’s influence. However, according to Andrews and Caren (2010), news media favour professional and formalised groups that employ non-confrontational tactics over volunteer-led, confrontational groups that advocate on behalf of topics beyond the mainstream.

## Emotions and Group Dynamics

Most literature on advocacy has mobilised structural theories of social movements, such as resource mobilisation, institutional perspectives, and political process (Almog-Bar & Schmid, 2014). Studies on group dynamics and the role of the emotions that arise during these interactions are still largely lacking in political advocacy research. Social movements scholarship has increasingly considered emotions but has predominantly done so to examine protests and contentious events (Goodwin and Jasper 2006; Van Ness & Summers‐Effler 2018). When emotions have been considered specifically in social movements’ political advocacy work, research has usually focussed on political communication (e.g. as in Bail et al., 2017, and Sanchez Salgado, 2018) rather than on group dynamics organising and enabling advocacy.

During our engagement in the field, we observed that group dynamics and related emotions had a significant impact on political advocacy. In line with our inductive approach, we decided to further explore the role of emotions in political advocacy and drew on the literature on emotions in group processes (Flam et al., 2005; Jasper, 2011; Goodwin et al., 2001). As King (2006, 876) explains, it ‘is evident that how emotions are constructed, managed, manipulated and reconstructed is important for understanding patterns of engagement in social movements by activists.’ For instance, the force of emotions such as joy, excitement, group belongingness, and solidarity have been found to sustain participation and commitment in social movements (Eyerman, 2005). Emotions help understand when social ties within networks afford particular emotional experiences that can support or hinder mobilisation (Van Ness & Summers‐Effler 2018). In addition, love, affection, and loyalty for other members can lead to action on behalf of the movement, while respect and trust for representatives of the movement can influence their legitimacy and credibility (Jasper, 2011). In this, the movement’s structure and formal or informal hierarchies may matter, with relations of status and power generating corresponding emotions (Goodwin & Jasper, 2006). In contrast, the lack thereof can contribute to a movement’s decline: Internal conflicts within a movement can induce anger and frustration, potentially leading to factionalism and even the disengagement of single individuals (Eyerman, 2005; Van Ness & Summers‐Effler 2018). Movement decline and destabilisation may indeed be explained by the absence or dysfunction of those diverse emotional processes and attachments that, in non-declining social movements, support a shared identity, reinforce social bonds, and generate emotional rewards for participation and costs for exiting (Van Ness & Summers‐Effler 2018).

These emotions are what Jasper (2011, 267) coined as ‘affective commitments or loyalties’ within movements, which he defines as ‘relatively stable feelings, positive or negative, about others […] such as love and hate, liking and disliking, trust or mistrust, respect or contempt’. Relatedly, Gould (2009) introduced the concept of emotional habitus, that is, ‘a social grouping’s collective and only partly conscious emotional dispositions, that is, members’ embodied, axiomatic inclinations toward certain feelings and ways of emoting. By directly affecting what people feel, a collectivity’s emotional habitus can decisively influence political action, in part because feelings play an important role in generating and foreclosing political horizons, senses of what is to be done and how to do it’ (ibid., p. 32). The emotional habitus operates beneath conscious awareness and provides the members of a social movement with shared emotional disposition. This involves not only which emotions are appropriate in given circumstances but also ways to express (emote) feelings and figure out what members are feeling (Goud 2009).

## Advocacy Spaces

Social movements can conduct advocacy work in different spaces (Giugni & Grasso, 2018). In her article ‘A guide for feminist advocacy’, Kristy Evans (2005, 14) encourages movements to reflect on the institutional spaces where relevant decisions are taken and in which spaces they can have the greatest impact. Political scientists have pointed out that the structure and scope of advocacy work often mirror the structure of the political system (Armingeon, 2002). For instance, in Germany, a federal republic, interest groups are typically also organised in a federal manner in order to have access to the right policy spaces (ibid.).

Agricultural movements and organisations in Germany can advocate at different levels: the supranational (i.e. EU), the national (federal) the state (i.e. Bundesländer), and the communal (i.e. municipalities). These levels have various degrees of decision-making power and roles when it comes to agricultural policies. Since agricultural policies are largely regulated at the European level via the Common Agricultural Policy (CAP), this level typically is considered to have considerable leverage (Feindt, 2009). However, it is challenging to advocate at the EU level, particularly for scarcely resourced grassroots movements. At the same time, there are possibilities to influence agricultural policy by advocating on the national (federal) level: The CAP is translated into national plans, which leave some room for country-specific implementations, such as the possibility to adjust direct payments for farmers (Henke et al., 2018). Despite this possibility, in the past, the German government made little use of its room for manoeuvring (Chemnitz et al., 2019)—notably, because the DBV has fiercely and successfully opposed mechanisms of reallocation (Chemnitz et al., 2019; Deutscher Bauernverband, 2021). In contrast, the German federal states use their leeway with regard to agricultural policy, leading to an ‘astonishing heterogeneity’ among the different federal states (Ewert, 2016, 253). This heterogeneity is also product of the advocacy work of agricultural associations: in those federal states where the federal farmer associations have a strong monopoly position, the productivist paradigm,^6^ which they defend, is particularly strong (ibid.).

## Research Design

### Positionality of the Authors

This research was part of a larger PhD project of the first author of this paper, which was carried out between 2019 and 2023. During her engagement with and participant observation of SOLAWI, she identified strong thematic alignment between her research interests and a side project of the network on political advocacy, which attempted to recompile crucial information on political advocacy. In fact, by chance both the first author and an employee of SOLAWI had been carrying out interviews on political advocacy with the same activists on similar issues. After several informal meetings, we decided to join efforts, notably in the form of co-designing and co-authoring this study. In addition to its academic contribution, this publication therefore seeks to produce ‘movement-relevant’ knowledge (Bevington & Dixon, 2005), which can instigate reflections on the potential, controversies, and difficulties around political advocacy work for SOLAWI specifically and agricultural grassroots movements more generally. This research is aligned with two core dimensions of scholar-activism; (i) the commitment to produce knowledge that is relevant and useful for SOLAWI as well as the involvement of activists in the process of knowledge production and (ii) having a strong relationship and political alignment with SOLAWI (Duncan et al., 2021).

As scholar–activists who have a strong relationship and alignment with SOLAWI and are committed to (co-)producing knowledge that is relevant and useful to its struggle, our hope is that this paper can provide reflections on the potential, controversies, and difficulties concerning advocacy work in the contexts of CSA networks and similar grassroots networks and inform future discussions regarding which forms of political advocacy may be continued. We are, however, not taking a general position on whether grassroots movements should engage in political advocacy. Rather, our starting point is the observation that grassroot movements, such as SOLAWI, have already been pursuing political advocacy as one strategy (among many), making it a relevant object of study that can inform these ongoing debates.

### Data Collection and Analysis

The data collection took place between April 2021 and January 2023. It followed standard research ethics procedures following the [obscured for peer-review] University code of conduct for academic practice. We drew on a diverse set of data including (i) participant observation in bi-weekly meetings over the span of approximately one year in the working group on politics and in the working group on organisational development, (ii) interviews with current and former active members of the network, and (iii) internal documents, including protocols of council and coordination meetings from 2018, when political advocacy work was discussed (see Appendix 1, Table 2 for an overview of the documents and the profile of the interviewees). We collected data on the formal network structures, but not on the informal ones (e.g. social relations beyond formal roles within SOLAWI), which may influence the SOLAWI’s ability, for example, to mobilise resources, reinforce organisational processes, and exert policy influence (e.g. see Diani, 1997; Saunders, 2013). In total, we conducted nine interviews, eight semi-structured, in-depth interviews, and one focus group interview with the working group on politics. The interviews lasted between 42 min and 2h09, with an average duration of 1h26. We complemented this data with background knowledge on political advocacy work more generally, which we obtained from participant observation at two three-day meetings of the SALSIFI project (Supporting Advanced Learning for Stakeholders Involved in Sustainable Food-systems Initiatives) on political advocacy work. SALSIFI is an Erasmus + project of the European Union running from September 2020 to August 2023. It is coordinated by the international CSA network Urgenci and brings together nine agri-food movements across Europe.

The first author of this paper analysed all data with NVivo, using open coding. After a first round of coding, the data was synthesised by the first and second author and collectively discussed with all co-authors. Subsequently, the first author recoded the results in several cycles of analysis, drawing on the theoretical framework presented in Table 1.

## Results: Political Advocacy as a Side Strategy

In what follows, we outline factors that have enabled and hindered political advocacy work. After a short overview of strategies and tactics adopted by SOLAWI across their advocacy work, the findings are structured using the theoretical framework (Table 1) on the network’s strategic orientation, its organisational structure, its ability to mobilise resources, the influence of emotions, and the spaces where advocacy is undertaken. While we draw on different instances of political advocacy, which have unfolded between 2018 and 2022, this study does not seek to reconstruct change processes; instead, our analysis dissects what has (or has not) worked with regard to political advocacy, thereby drawing lessons for the future.

## Strategies and Tactics: Overview

SOLAWI has employed various outsider and insider tactics to pursue its goals. However, because of limited resources, the network was forced to prioritise these tactics. This prioritisation, which was co-decided by the council and coordination, is re-negotiated every two years, following the election of a new council. Beyond this, the CSA network did not have a long-term strategy.

By and large, the network prioritised outsider tactics. Supporting a prefigurative politics, the network provided several services that aimed to spread the CSA model and consolidate existing initiatives (Doc-1). This included providing support and advice for individual CSA initiatives in their foundation phase and beyond via webinars, consultations, referral to counsellors, and shared support materials (NWSL, 2021). The network further facilitated mutual learning, knowledge exchange, and networking between CSA initiatives by providing spaces, such as the bi-annual network meetings, regional meetings, email lists, and online collaboration platforms. More conventional outsider tactics included the participation in protests and petitions, most notably the annual large-scale manifestation *‘Wir haben es satt!’* (‘We are fed up!’), which calls for a fundamental transformation of the agri-food system towards a small-scale, environmentally friendly, and globally just agriculture.

By contrast, insider tactics—namely, carrying out advocacy and targeting political decision-makers—only played a secondary role for the network (Doc-1). The low prioritisation of political advocacy was reflected by the lack of allocated resources for that purpose. Consequently, with the exception of the aforementioned government agreement, for which a delegation of SOLAWI advocates proactively contacted politicians, the network largely carried out reactive political advocacy. That is, while it sought to respond to requests from authorities and politicians and support CSA initiatives that invited politicians for farm visits (see Fig. 2), it did not proactively reach out to well-positioned policy makers. Since there was no dedicated person or team in charge of carrying out advocacy, the requests were answered by employees or activists who were interested in and available for conducting political advocacy. Nonetheless, the low prioritisation of political advocacy and the choice to focus on reactive advocacy were hardly sufficient to induce changes in agricultural policies or to receive institutional support.

A further, and perhaps more fundamental, barrier against the use of insider tactics was the reservations that some members held against engaging in political advocacy. In particular, the internal discussions in the aftermath of being mentioned in the government agreement revealed a twofold concern regarding the possibility of advocating for and receiving federal funds. Some members feared that the network could become financially dependent on political parties (Doc-2); they reasoned that if the network was to receive a large sum from the government for a limited time frame and if they were to use the funds to enlarge their staff, they could run into difficulties once the funding ended as they would be no longer able to pay their employees. Another related concern was a fear of loss of autonomy; some activists associated receiving institutional support with no longer being able to independently decide how and what financial resources can be used because of the official requirements associated with the reception of governmental funds (Doc-2).

## Organisational Structure: Increasing Professionalisation and Agility

The network has become increasingly professionalised over the past 5 years. This change is noticeable in the steady increase in employees—at the time of writing, there are six paid (part-time) staff members and three ‘mini-jobbers’ (i.e. employees who earn less than €520/month). There has also been an increase in organisational development efforts, which entailed redefining decision-making structures inspired by sociocracy and restructuring the roles of the different organs of the network. As part of this process, the role of the council was redefined. Instead of taking fundamental strategic and political decisions, it began to oversee the operational activities that the employees and the coordination carried out, ensuring their compliance with the network’s jointly established goals, values, and strategies. The adjusted role of the council promised more agility and faster decision-making since previously, the tasks and responsibilities of council members frequently exceeded their time capacities. This shift may ultimately enhance the network’s ability to engage in political advocacy. When CSA was mentioned in the government agreement, several advocates pointed out that slow decision-making processes significantly hindered their work and foreclosed the possibility of seizing further, more substantial gains and support (I-1; I-2).

While the network had extensively worked on its internal organisation, there were no clear structures or procedures for nominating or legitimising political advocates. In the past, activists received (i) long-term mandates for political advocacy because of their personal contacts to politicians or (ii) ad hoc mandates after having received an invitation to speak at an official event. These mandates were rather fuzzy; it was not specified how extensive the mandate was, what positions and demands the activists should voice, in what way and with what frequency they ought to report back to the general network, nor under what conditions the mandate would lose its validity. On one occasion, these uncertainties delayed and thereby hampered efforts to advocate, since a more specific mandate was needed to continue the ongoing negotiations (I-2).

All in all, the broad base of the network’s members hardly had a say in political advocacy. While members provided considerable financial stability to SOLAWI (NWSL, 2022), they were not directly involved in deciding how and for which activities the finances were allocated. Instead, the prioritisation of activities and tactics fell under the responsibility of the council and coordination, and the broad member base influenced them only very indirectly via the election of the council’s members. Consequently, it is unclear to what extent the prioritised tactics and activities matched the needs and wishes of its members—in other words, whether political advocacy would have received more importance when taking into account the members’ preferences. In fact, anecdotal evidence points to a few CSA initiatives that decided to join the *Arbeitsgemeinschaft bäuerliche Landwirtschaft*^7^ (AbL—the German peasant organisation) instead of SOLAWI since AbL’s advocacy work was more professional (I-3).

Those members who wished to do political advocacy could nonetheless take initiative, as the network left ample room for self-organisation. The working group on politics, established in 2022, bridged and coordinated different network activities related to political advocacy, alliance work, and public relations. It brought together actors involved in political advocacy from the federal, state, and supranational levels. This working group’s principal aim was to raise awareness within the network regarding the potential benefits of political advocacy for CSA and to develop a list of demands that advocates could use when interacting with policy-makers. Furthermore, several members of the working group had participated in meetings with politicians organised by local CSA initiatives in order to support those initiatives (I-3).

## Resources: The Bottleneck for Political Advocacy

Limited resources, in particular material and human resources, significantly hampered the political advocacy of the network, despite the successful mobilisation of moral and socio-organisational resources.

With a yearly financial budget of approximately €260,000, the network was underfinanced and, as mentioned above, forced to prioritise the activities it engaged in. The largest and most stable form of revenue for SOLAWI in 2022 was membership fees (42%), followed by donations (27%), and grants (8%) (NWSL, 2022). To a large extent, the budget financed the paid staff positions of the network. However, since political advocacy did not fall under the prioritised activities, only a mini-jobber, whose position was created through funds of the SALSIFI project, focussed on political advocacy. The other employees, who all faced a high workload due to the increasing responsibilities, tasks, and challenges that accompanied the growth of the CSA network, only engaged with political advocacy to a small extent (I-4; I-5). Nonetheless, some key activities of employees overlapped with political advocacy—most notably, networking, public relations, and event organisation.

Because of the lack of funding for political advocacy, most activities relied on voluntary work. However, there were a number of challenges associated with relying solely on volunteer work. First, similar to the staff members, most volunteers experienced a very high workload, as they were often also heavily involved in their local CSAs and other community projects. Second, while political advocacy was demanding in terms of knowledge and time requirements (I-5; see also Appendix 2 for an overview of key skills for political advocacy), the volunteers often had a diverse set of skills, time availability, expertise, experiences, and social ties and, consequently were differently equipped to carry out political advocacy. An interviewee with extensive experience in advocacy therefore voiced the need for paid personnel dedicated to political advocacy (I-2). Third, the reliance on volunteer work for political advocacy turned out to be risky when advocates disengaged from the network in 2020, resulting in a tremendous loss of knowledge and skills and a temporary halt of advocacy activities (I-5). That incident further highlights another problem: the concentration of knowledge within a few activists and a lack of knowledge transfer. With hardly any internal documentation of past advocacy work, it was difficult and time-consuming for new staff members and volunteers to recompile the necessary information and contacts to continue political advocacy. This challenge became evident during the organisation of an event directed at the intersection of politicians, civil society, and scientists since there was no documentation of the organisation of previous events nor contact lists of relevant actors who should be invited (I-3). To some extent, the SALSIFI project remedied this situation by providing the network with funds to conduct interviews with those activists who, in the past, had been active in advocacy work for the network. The project not only took stock of the existing knowledge on political advocacy for CSA but also made it available to interested activists in the form of an online course.^8^

Beyond material and human resources, the network successfully mobilised moral resources. First, the network strategically drew on research to work with politicians to legitimise the CSA model and its socio-economic and sustainability potential (Doc-7). Second, in 2018–2019, the network mobilised politicians from different parties (i.e. the Green Party, the Social Democratic Party [SPD], and CDU) for their cause. For instance, the network arranged a meeting with an MP of the SPD who was already knowledgeable of and sympathetic to the CSA model, to jointly strategise how CSA could receive support (I-7). Afterwards, the MP publicly expressed his regard for CSA during a speech at the German Parliament (I-5; see also Deutscher Bundestag, 2018) and released a supportive statement on his webpage (Spiering, 2018). The MP, who had little formal power, further helped the network by asking the Chief Secretary of Agriculture, an influential politician of the CDU, to meet with a delegation of SOLAWI and to assess possibilities for institutional support (I-1). While the latter showed general interest in and openness towards the CSA model, the meeting only resulted in a small grant for an educational project for SOLAWI, since, by then, most relevant funds of the ministry had already been used up (I-6). At the time of writing, most of the MPs at the national (federal) level who were supportive of CSA in 2018–19 had retired from politics, weakening the network’s moral resources.

Finally, the network mobilised a number of socio-organisational resources in the form of social ties and allies. Because one advocate had personal contacts to politicians, political advocacy was greatly facilitated (I-2). In some instances, the network was able to capitalise on these social ties and turn them into organisational ties. For example, in January 2023, SOLAWI co-organised, for the third time, a symposium on CSA with the foundation of the Green party, even though no personal contacts had remained. In addition to reviving old ties, since 2022, the network has been building new connections and contacts—in particular, to high-level employees of the Federal Agency of Agriculture and Food (‘Bundesanstalt für Ernährung und Landwirtschaft’).

A strong asset for conducting political advocacy was the network’s alliances with likeminded organisations, many of whom allocated significant financial and personal resources to political advocacy. An important ally of the network was the AbL, which is like a ‘big sister’ to SOLAWI (I-8). The AbL devotes significant resources to, and, with its expertise, has established professional structures for political advocacy, both on the national (federal) and the state level. Because the AbL’s goals overlap with those of SOLAWI (both call for substantial support for smallholder farmers), some activists have suggested to intensify their collaboration (I-3). Moreover, the network is part of the ‘Agrarbündnis’, an agricultural alliance consisting of environmental and alternative agri-food associations. In addition to their advocacy activities and campaigns, the Agrarbündnis issues a yearly report on the state of agriculture in Germany and Europe to which SOLAWI has repeatedly contributed to (see e.g. van Elsen & Kraiß, 2012; and Kapusta, 2023). Additionally, SOLAWI is also part of Urgenci, which, at the request of SOLAWI activists, launched two projects on political advocacy that contributed to knowledge exchange and capacity building for political advocacy. Lastly, being well-networked and having allies can create unexpected opportunities for advocacy around CSA. For instance, the environmental organisation BUND (Friends of the Earth Germany) chose a CSA farm as a location for an inauguration event on organic agriculture, which was attended by the federal state minister of Mecklenburg-Western Pomerania. Shortly after, the minister invited all CSA initiatives of the federal state to the Ministry of Agriculture to continue the conversation and explore how the ministry could support CSAs better.

## Emotions and Group Dynamics: The Elephant in the Room

Emotions and internal group dynamics had significant impact on the network’s ability to carry out advocacy work. We found that both a lack of trust in advocates and a lack of visibility and valorisation of advocates can lead to frustrations and a loss of motivation and thereby hinder political advocacy. The internal discussions of how to proceed after being mentioned in the government agreement and the negative emotions this triggered illustrate these points clearly. During a council meeting in 2018, shortly before an upcoming negotiation with an MP, the delegation of advocates was confronted with questions and concerns by other council members who asked to bring an additional council member to the negotiation (Doc-2). This request, which was interpreted as a sign of mistrust, offended the delegation, which quickly affirmed that the negotiations would naturally be carried out in the best interest of SOLAWI and that there was no need for an additional member to join and ‘control’ them (Doc-2). Instead, to allay the doubts and mistrust, the delegation proposed that the council should jointly develop a concrete list of demands, which they could take to the negotiations, thereby ensuring that the negotiations reflect the wishes of the council. They further promised to consult with the council when decisions were imminent—a procedure that was eventually adopted. However, the mistrust was not resolved, essentially remaining the elephant in the room (Doc-2; I-2). Potential explanations for the mistrust voiced by activists ranged from previous bad experiences such as the abuse of mandates in other organisations (Doc-2) to the perception of one advocate not being politically neutral and impartial, due to his double role (besides to his volunteering for SOLAWI, he worked for an MP) (I-2; I-9).

Another source of frustration was the lack of visibility and limited valorisation of the advocacy efforts. Political advocacy is a laborious task, and some volunteers spent up to 15 h per week advocating for SOLAWI (I-1); however, these efforts at times remained hidden. For instance, to inform its members of being mentioned in the government agreement, only a brief statement was circulated via the newsletter: ‘The [CSA] network was mentioned in the government agreement’ followed by a quotation from the government agreement (NWSL, 2018). The statement entirely concealed the efforts of advocates to SOLAWI members who are only sporadically active. In addition to the lack of visibility, there was also a lack of valorisation. Several activists reported that they did not feel sufficiently appreciated for their volunteer work by other SOLAWI members, leading to frustrations and, eventually, their disengagement from the network (I-1). Such a lack of valorisation was also rooted in the prioritisation of different strategies and activities by activists: While for some, political advocacy was critical to induce change in the agri-food system, others did not view it as essential or productive. Altogether, these instances showed that negative emotions and knotty group dynamics can render political advocacy particularly difficult and even lead to the disengagement of activists (I-1; I-2).

Despite those past issues, trust in and valorisation of advocates have ceased being major issues of contention during the rebuilding of political advocacy within SOLAWI in 2022–2023. Moreover, driven by the appointment of a new public relations officer, the visibility of advocacy efforts has also increased. Activists who carried out political advocacy efforts did so only sporadically, as one of many activities, and thus were less emotionally invested than the former fixed delegation of advocates, who approached politicians on a regular basis. Nonetheless, members of the working groups on politics voiced that, at times, there was a tendency among network activists outside their group to give input on areas outside of their area of responsibility. Such unsought advice can be tiring for the person in charge and lead to irritations (I-3). The working group pleads that trust is not only a matter of verbal expression; instead, it needs to be lived and practiced, for instance, by refraining from interfering (I-3).

## Advocacy Spaces: Moving From the National (Federal) to the State Level

Over time, political advocacy shifted from primarily the national (federal) to the state level (see Fig. 2). From 2018 to 2019, because of pre-existing personal contacts of one advocate to an MP, political advocacy was mainly conducted at the national (federal) level, targeting politicians working on agricultural topics. The advocacy efforts primarily aimed to raise awareness for CSA and to mobilise institutional support, while more specific, content-related demands largely exceeded the capacities of SOLAWI (I-2). The lack of a list with demands for agricultural policy changes clearly illustrates this point. Content-based advocacy was therefore limited to sharing, supporting, and signing statements, petitions, and demands of their allies—for instance against seed patents^9^ and the privatisation of public agricultural land in Eastern Germany.^10^

After personal changes within the network in 2020, most advocacy efforts were located at the federal state level. In total, in five of the 16 federal states, some form of advocacy work was undertaken (see Fig. 2). In fact, the, until 2021, largely neglected state level yielded new opportunities for SOLAWI. According to one activist with a law background, many agricultural policies that have hindered CSA initiatives are regulated at the federal state level, such as building permits for agriculture land, which are exclusively issued to agricultural holdings. This regulation has inhibited many CSA initiatives organised as associations from building structures, such as foil tunnels, on their land plots (personal communication 21 st January 2023). Additionally, the Chambers of Agriculture, which promote and support farmers and which are responsible for agricultural vocational training (a major concern of SOLAWI), are exclusively located on the federal state level.

However, carrying out political advocacy at the *federal* state level is not easily put into practice by a *national* network. Consequently, at the time of writing, the network is in the process of appointing several representatives for a number of federal states. These representatives can serve as contact persons when receiving invitations from federal politicians or authorities.

## Discussion: Internal Organising Matters for Political Advocacy

**Fig. 3:**
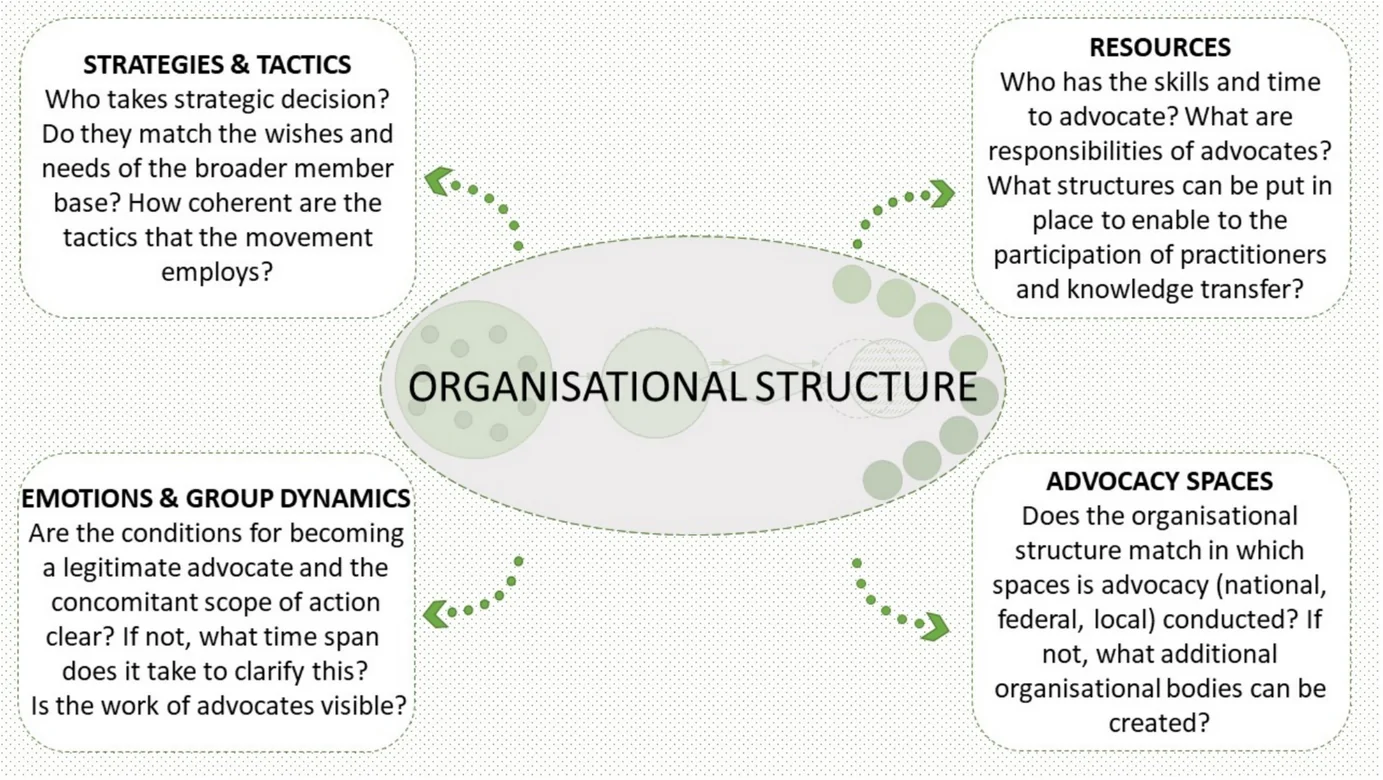
Questions arising from the interlinkages between the network’s organisational structure and remaining dimensions of the framework

In what follows, we discuss the intersections between the dimensions of the framework that, in our opinion, have the most direct practical relevance for SOLAWI. While we acknowledge that political influence is not something that movements ‘can simply provide, pizza-like, for themselves’ (Amenta et al., 2010, 96), we nonetheless argue that the network’s internal organisation intersects with their strategies and tactics, ability to mobilise resources, emotions and group dynamics, and advocacy spaces (see Fig. 3). We discuss how these intersections affect the network’s ability to advocate, including possibilities to organise more effectively for political advocacy. Thereby, we point out a number of open issues practitioners can take into account.

## Organising and Tactics: Exploring Member’s Participation

With regard to the interconnection between SOLAWI’s organisational structure and its strategies and tactics, we have observed potential tensions concerning the prioritisation of tactics. As explained above, it is unclear to what extent the prioritisation of tactics, including the low prioritisation of political advocacy, reflects the needs and wishes of the broader member base. A survey by Lapschieß and Degens (2023) on the perceived importance of the activities undertaken by SOLAWI suggests otherwise: Political advocacy was one of the activities that established CSA initiatives valued most; over 60% of all respondents considered advocacy important, with only a few other activities receiving more approval—such as providing knowledge resources and a platform for mutual exchange, consulting services (e.g. for taxes and organisational development for CSA initiatives), and the provision of training on agricultural and economic topics. However, because of the relatively small sample size (only 10% of all CSA initiatives participated in the survey), those results need to be carefully interpreted, and the network should consider further exploring the needs of its members. Given the heavy workload of both activists and employees, it is important to consider how this exploration can be organised in a way that allows for ample participation without requiring too much work and time. Already existing (offline) formats and spaces, such as the network meetings, a space that gathers around 100 CSA activists, could serve as a first step to assess how well the network’s prioritisation matches the wishes of the member’s base.

The representation of the members’ views and needs is particularly salient considering the rapid membership growth of SOLAWI. The network faces the challenge of preserving its grassroots character despite its growth. It must consider how the network can defy the risk which so many movements and advocacy groups are susceptible to—namely, that a small group takes decisions on behalf of the whole movement (see Andrews & Edwards, 2004 and; Foley & Edwards, 2002 on oligarichisation tendencies in advocacy organisations). In light of the increasing digitalisation and growth of SOLAWI and the geographical size of Germany, it is difficult for CSA initiatives from all over the country to attend the network meetings. In response to this challenge, digital platforms could become ‘decisive tools for mobilizing, for organizing, for deliberating, for coordinating and for deciding’ (Castells, 2015, 257).

In addition, to maintain its grassroots character, the network leaves ample room for self-organisation beyond the officially prioritised tactics and strategy and concomitant use of resources. As such, members can, in a decentralised and unbureaucratic manner, pursue activities they are passionate about, including political advocacy, on their own behalf as volunteers. The tactics that the network employs with regard to political advocacy are therefore, to a large extent, the result of a variety of ad hoc or reactive actions of individuals, and they are not necessarily aligned. To align a movements tactics (i.e. their means) and goals, developing a long-term strategy is necessary (Doherty & Hayes, 2019), yet such a strategy is currently missing for SOLAWI. Mid- and long-term thinking could therefore help activists to strategise freely, without the constraint of limited financial resources and may entail moving from reactive to proactive political advocacy, as well as clarifying (i) with which actors the network wishes to engage with and to what extent, (ii) for which advocacy spaces demands should be formulated and placed, and (iii) what alliances should be strengthened.

## Organising and Resources: Ambiguities Around Responsibilities and Legitimacy of Advocates

The organisational structure of SOLAWI intersects with the network’s ability to mobilise resources in at least three ways. First, regarding who can advocate and whose voices are represented, there is a lack of organisation in the decision-making process. Consequently, as shown in this study, political advocacy is highly dependent on human resources, notably volunteers, and carried out only if and as long as individual activists step forward (or react to external mobilisation).

In light of the high time investment required for political advocacy (Almog-Bar & Schmid, 2014), the network is in need of establishing mechanisms that help with managing and distributing the high work-load of volunteers. This need includes defining the degree of accountability and reporting back which can be expected of volunteers—in particular of farmers, who already have a high workload. The difficulty for advocates to report back in full transparency in light of ‘time and other human constraints’ also applies to agricultural grassroots movements more generally (Hitchman, 2014, 13). What can SOLAWI learn from these movements and their efforts to reduce the workload on farmers and gardeners who advocate? For example, the CSIPM (Civil Society and Indigenous People Mechanism^11^) teams up food producers who are eager to be involved in advocacy work with volunteers that have (potentially) more time and can take over background tasks such as reporting back after advocacy events, taking minutes, and writing draft contributions on the basis of conversations with food producers (see Claeys & Duncan, 2019). While this type of work-sharing comes with its own limitations—in particular, the concentration of expertise and power within a few dedicated individuals (ibid.)—it can enable food producers to adopt the advocate role. It is important that farmers take up the role of advocates, since their personal experience with the difficulties of farming and running a CSA renders them particularly powerful and legitimate advocates (see also Appendix 2).

Additionally, drawing on her experience and engagement with transnational advocacy for agricultural movements, Hitchman (2014) argues that it is vital that advocates (especially those who are not practitioners) remain connected to the grassroots level to be knowledgeable of the concerns and pressing issues that movement practitioners face and receive input for policies (see also Claeys & Duncan, 2019 on the need and difficulty of ‘grassrootifying’ struggles to ensure legitimacy and adequate representation). Thus, SOLAWI could establish procedures that help volunteers to collect the views of the member base.

Furthermore, because of a lack of resources, the network has not yet defined a set of criteria regarding which circumstances and what topics committed activists can or cannot legitimately advocate and represent SOLAWI in the presence of policy-makers. To compensate for this lack of clarity, the network has issued temporary ad hoc mandates and has asked activists to only speak in their own name when engaging in advocacy. However, this coping strategy has significant drawbacks. If political advocacy for CSAs is not shaped by a collectively negotiated politics of the network, but by single advocates, this could lead to the (co-)existence of potentially contradictory demands. The CSA network is, after all, heterogenous and has different factions; some position themselves as an actor of a societal transformation more broadly, while others push for the safeguarding of small-holder agriculture (Spanier-Guerrero Lara & Feola, 2024). This heterogeneity results in different political agendas; for example, the former openly questions the viability of private property and strives for collectivising land and means of production, while the latter are often land-owners themselves.

Additionally, it is uncertain whether policy makers and administrative elites, who are not familiar with the structure of SOLAWI, will understand whether CSA advocates are speaking on their own behalf or on behalf of the network, especially if this role keeps changing. These practical difficulties around questions of legitimacy and representation are fundamental to any convergence process in (agricultural) grassroots networks (Claeys & Duncan, 2019), and they therefore need more attention from scholars and activists alike.

Third, a lack of internal organisation—in particular, missing structures for knowledge transfer and sharing—temporarily led to the concentration of knowledge within a few activists and consequently hampered political advocacy. Setting up organisational structures that facilitate knowledge sharing—for instance, by a meticulous documentation of ongoing political advocacy processes or by setting up trainings, such as the SALSIFI project—is an important step to decrease dependency on individuals and prevent new staff members and volunteers from having to repeatedly recompile the necessary information, skills, and contacts for carrying out advocacy. This need for knowledge sharing echoes the findings of Onyx et al. (2010), who stressed the necessity to organise training sessions in self-advocacy skills as well as knowledge and skill sharing for effective advocacy.

## Organising and Emotions: Creating the Basis for Political Advocacy

While the importance of emotions in studies of political advocacy and its interrelation with a movement’s organisational structure have been overlooked by extant research, the case of SOLAWI provides empirical evidence for how a group’s internal structure can adversely affect members’ emotions and group dynamics. Earlier research discussed status and power relations (Goodwin & Jasper 2006), and factionalism (Van Ness & Summers‐Effler 2018), among other factors that may generate fracturing and destabilising emotions within social movements, leading to demobilisation or abandonment of some activists. Complementing those insights, our study suggests that unclear organisational structures and recurrent discussions on whether activists are legitimate advocates are likely to cause frustrations and aggravate already existing tensions among the activists. These feelings may, in the worst case, lead to the disengagement of some individuals whose status as legitimate advocates for the movement was repeatedly challenged by other activists (see also Eyerman, 2005 on the decline of movements as a consequence of anger and frustrations among activists). Therefore, clearly defining the roles and responsibilities is vital to ensure that the motivation of advocates is not compromised by long and complicated internal negotiations regarding whether and what topics they can(not) advocate on.

Furthermore, ensuring the visibility of advocacy efforts—for instance, by appreciatively reporting on advocacy activities—can nurture the positive affective bonds and solidarity to sustain collective action (Jasper, 2011). First, this form of visibility may give advocates the feeling that their efforts are valued by the broader movement, which may be particularly relevant in light of the factionalism within SOLAWI (Spanier-Guerrero Lara & Feola, 2024) and the concurrent rise and fall of the status and power of activists (Kemper, 2001). Second, providing additional information may enhance transparency and, concomitantly, the trust that members have towards advocates.

Cutting across the points above, we observe the need for SOLAWI to reflect on its emotional habitus (Gould 2009): what emotional norms, shared ways of feeling and of expressing emotions are considered acceptable in connection with political advocacy work and its inherent outward-looking function. As suggested by Gould (2009), while the emotional habitus structures what a social movement members feel and how they express their feelings, individuals with different personal histories may be shaped differently by such habitus; and while the emotional habitus provides preconscious means to interpret each other’s affective states within a movement, some activists may experience feelings that are considered unacceptable and not conform by suppressing them. These situations may generate social dynamics of conflict and the departure of some members.

## Organising and Advocacy Spaces: Identifying (Mis-)matches

How a movement is organised influences the spaces in which political advocacy can be fruitfully conducted. That is, political impact is more likely when movements adjust their organisational structure and tactics to match the institution they seek to influence (Amenta et al., 2010).

Being situated in a four-level policy field, namely the EU, federal, state, and communal level, opens, at least in theory, different entry points for advocacy. As discussed in the previous sections, SOLAWI is a rather young and under-resourced organisation, which, in order to be effective, needs prioritise and focus their advocacy efforts, particularly given that some levels advocacy efforts are likely more resource-intensive than others. Thus, while the EU level, with its protective policies and area-based direct payments, largely determines the viability of farms, SOLAWI does not directly seek to influence EU-policies. In comparison to large, powerful farmers associations, which invest considerable time and financial resources to advocacy, SOLAWI is ill-equipped to lobby for their demands. Instead, URGENCI, the international network of CSAs, fills in this role on behalf of all European CSA networks.

Representing CSA initiatives from all over Germany, SOLAWI is well positioned to conduct political advocacy on the national (federal) level. At the same time, the network is still a relatively small actor in the German agri-food system and lacks resources, contacts, and expertise to advocate on its own. Small(er) movements tend to form alliances and join political advocacy campaigns of larger movements, which push for more fundamental changes (Onyx et al., 2010). Similar to the UK CSA network (see Bonfert, 2022), SOLAWI already has established alliances at the national (federal) level with other agricultural grassroots movements, notably the AbL and Agrarbündnis. Thus, strengthening these already existing alliances and thereby supporting systemic changes in the agri-food system are necessary to move from building a supportive environment for CSAs to bringing about a paradigm change in agriculture more generally (see Spanier-Guerrero Lara & Feola, 2024 on the benefits for CSA to build alliances with movements that demand structural changes).

Furthermore, as the findings show, the network is increasingly shifting its focus of advocacy to the federal state level. Since SOLAWI, contrary to other agricultural advocacy organisations such as the DBV (Feindt, 2009), does not (yet) have any federal divisions, its internal structure does not mirror the political system (Armingeon, 2002); consequently, it is not fit to advocate on the federal state level. This limitation is supported by observations that the organisational structure of advocacy organisations differs significantly depending on whether they operate on the national (federal), state, or communal level (Andrews & Edwards, 2004).

However, adjusting and expanding the internal structure, including nominating representatives for conducting advocacy in the federal states, is complex. It requires a two-fold legitimisation process: The network needs to approve the federal advocacy representatives, and the local initiatives of the respective federal state, too, need to give input into who can advocate on their behalf in the future. Nonetheless, this effort may be well worth it; engaging on the federal state level could open new possibilities to SOLAWI. In particular, since many agricultural policy decisions are taken at the state level, such involvement could complement SOLAWI’s focus on advocating for institutional support, with efforts to place concrete demands for agricultural policies. As pointed out by Ewert (2016), the federal states shape the realities of food producers in several ways. Besides providing financial incentives, such as agri-environmental schemes, the federal states also have legislative power over a range of topics, including agricultural and environmental protection laws, and they shape agricultural vocational training via the Chambers of Agriculture. Thus, the federal states are most relevant for pushing for changes which are specific to CSA. For example, the case of building law, mentioned above, illustrates how political advocacy in the respective federal states could contribute to the removal of legal and administrative barriers which render the everyday practices of CSA gardeners particularly difficult.

## Conclusion

This study found that SOLAWI’s internal organisational structure (or lack thereof) influences the strategies and tactics, resources, emotions and group dynamics, and advocacy spaces in various ways and thereby can enable or hinder political advocacy. Because of the limited engagement of the broad member base in deciding which strategies and tactics are prioritised, it is unclear whether the low prioritisation of political advocacy reflects the views and wishes of SOLAWI members. In light of its limited financial and human resources, the network needs to clarify the responsibilities and legitimacy of advocates and to develop procedures that allow food producers to carry the voice of the movement. This analysis further showed that negative emotions, such as frustration, lack of trust, and not feeling appreciated (which typically are overlooked in studies on political advocacy), can be partially traced back to unclear role descriptions. These emotions undermine advocates’ motivation to further pursue advocacy. Finally, while the political advocacy efforts of SOLAWI have shifted from the national (federal) to state level, thereby opening new spaces to influence agricultural policies, adjusting the network’s internal structure brings new challenges in terms of legitimacy. We therefore conclude that movements and practitioners wishing to engage in political advocacy may benefit from paying due attention to the organisational structure of their movement, including some of the tensions and questions that we raised in the previous section (see Fig. 3).

This paper primarily makes an empirical contribution that has the ambition to inform advocacy work, while it offers scholars of political advocacy insights into poorly documented organisational dynamics and a movement that has been overlooked in the political advocacy literature. In doing so, this paper also makes the case for bringing the role of emotions into analyses of political advocacy, which have been dominated by structural theories, thus offering an enriched perspective—crystallised into a framework (Table 1)—which promises to have wider applicability in political advocacy research. Future research on the political advocacy of SOLAWI and other similar social movements may fruitfully adopt a longitudinal design to discern the impact of organisational development on political advocacy and explore not only the formal roles and relations but also the informal networks within and beyond the social movement boundaries that are likely impacting its capacity to develop and sustain political advocacy.

## Data Availability

The interview data that support the findings of this study are not publicly available. The documentary data analysed during this study are listed in this published article (Appedix 1) and links are provided.

## Appendix 1

**Table 2:** Profile of interviewees

Acronym	Role (at the time of being interviewed)	Duration
I-1	Former board member and advocate	2h09
I-2	Former board member and advocate	46 min
I-3	Focus group: Members of the working group on politics composed by staff members and active advocates	2h00
I-4	Staff member	1h44
I-5	Staff member	1h02
I-6	Former advocate, staff and council member	1h44
I-7	Former board member and advocate	1h24
I-8	Board and council member	1h21
I-9	Staff member	42 min

Overview of Documents.

Doc-1: NWSL, 2021. Prioritisation of the network’s goals and targets, June 2021. *Unpublished document*.

Doc-2: NWSL, 2018. Protocol of the council meeting 30.11.-02.12.2018. *Unpublished document*.

Doc-3: NWSL, 2018. Protocol of the conference call of the coordination meeting 23.08.2018. *Unpublished document*.

Doc-4: NWSL, 2018. Protocol of the conference call of the working group on organisational development 14.09.2028. *Unpublished document*.

Doc-5: NWSL, 2018. Protocol of the conference call of the coordination meeting 20.09.2018. *Unpublished document*.

Doc-6: NWSL, 2018. Protocol of the conference call of the coordination meeting 10.11.2018. *Unpublished document*.

Doc-7: NWSL, 2020. „Welche Möglichkeiten gibt es bei Wertschöpfungsketten und Vermarktungsstrukturen, um die Stellung der Landwirtinnen und Landwirte zu stärken? Anhörung von Sachverständigen Enquetekommission V ‘Wertschöpgunsketten Und Vermarktungsstrukturen.‘“ https://www.landtag.nrw.de/portal/WWW/dokumentenarchiv/Dokument/MMST17-3335.pdf.

Doc-8: NWSL, 2023. Stabil in die Zukunft. Dokumentation des Frühjahrstreffen 2023. https://www.solidarischelandwirtschaft.org/fileadmin/media/solidarischelandwirtschaft.org/Veranstaltungen/Netzwerktreffen/2023/FT23/Dokumentation_FJT23.pdf.

## Appendix 2

Box 1: Overview of key skills and abilities of advocates based on expert interviews I-1; I-2; I-6; I-7.

**Table:**

Skills, abilities, and professional experience of advocates *Tailoring discourses to target audience*: Which topics and key words resonate with politicians depends on their background, i.e. their political party and area of expertise. For instance, politicians of the Green party may be open to CSA when it is presented as a form to ensure the survival of small-scale peasant agriculture, environmental protection, or as participatory civil society initiatives. In contrast, politicians of the CDU may more inclined to support CSA when framed as safeguarding traditional family agriculture and stimulating rural areas *Using formal and informal channels:* Next to formal requests and official, written demands, political advocacy fundamentally relies on seizing and participating in informal moments and meetings. For instance, approaching an MP after the official panel discussion over drinks paved the way for getting CSA into the government agreement. Contrary to formal channels, informal channels heavily rely on personal continuity on both sides *Assessing role of politicians:* Advocates need to have knowledge about the different responsibilities of politicians and identify and target those with similar values and decision-making power. Thus, advocates should not shy away from contacting politicians with high functions. If these are not ideologically close to the values of CSA, advocates can use brokers, i.e. politicians that support CSA and have good contacts to the politicians in question, to get access to decision-makers (see also moral resources below) *Recognising ‘windows of opportunity’:* Awareness of key timelines of parliamentary and agricultural policy—that is, when are things debated or decided on—is important. For instance, co-organising an event during the alternative Green week, which precedes and opposes the International Green week, the world’s largest (conventional and productivist) agricultural fair, enabled a high-attendance from politicians and media coverage *Showing presence and persistence:* To make contacts and make CSA as a concept more widely known among politicians, it is important to appear at different events related to agriculture, food systems, and regional development. When reaching out to politicians, always follow up emails with calls. This requires tolerance to frustration, as often there is no immediate positive response and advocates need to present their concerns multiple times *Having ‘hands-on experience’ with farming*: Stressing first-hand experiences with and the every-day difficulties in agriculture tends to convince policy makers better than reporting dry facts and statistics that, e.g. centre the vanishing of peasant agriculture. Additionally, personal narratives can increase the credibility of advocates and relevance of their demands in the eyes of politicians *Sharing insights from the broader CSA movement*: Narratives become more powerful when advocates complement their own experience by referring to the situation of other CSA projects, as this signals to politicians that CSA is a widespread phenomenon and that the topics discussed are also relevant to other actors	

## Footnotes

1 https://www.solidarische-landwirtschaft.org/startseite.

2 Interview #2, henceforth I-2.

3 See also: https://communitysupportedagriculture.org.uk/.

4 Last accessed: 08–03-2022.

5 See also subsection on human resources below.

6 See also Lang and Heasmann ((Lang, and Heasman, 2015)) on the origins and dynamics of the productionist paradigm globally.

7 See also https://www.abl-ev.de/start.

8 See https://hub.urgenci.net/salsifi-course-program/.

9 See https://www.solidarische-landwirtschaft.org/aktuelles/news/news-detail/online-petition.

10 See https://www.no-patents-on-seeds.org/index.php/de/petition.

11 The CSIPM is an autonomous part of the UN Committee on Food Security. For more information see: https://www.csm4cfs.org/what-is-the-csm/.

## Declarations

The research presented in this paper involved primary data collection from human research participants.
